# Fluoride levels in river water from the volcanic regions of Cauca (Colombia)

**DOI:** 10.1007/s10661-022-09999-2

**Published:** 2022-04-05

**Authors:** Inés A. Revelo-Mejía, Robinson Gutiérrez-Idrobo, Vilma A. López-Fernández, Alejandra López-Rosales, Francy C. Astaiza-Montenegro, Leomary Garcés-Rengifo, Paola A. López-Ordoñez, Arturo Hardisson, Carmen Rubio, Ángel J. Gutiérrez, Soraya Paz

**Affiliations:** 1 Faculty of Odontology, Universidad Antonio Nariño, PopayánBogotá, Cauca Colombia; 2 Faculty of Odontology, Dentist Research Group, Universidad Antonio Nariño, PopayánBogotá, Cauca Colombia; 3 Faculty of Odontology, Dentist Research Group, Municipalities of Coconuco and Puracé, Universidad Antonio Nariño, PopayánBogotá, Cauca Colombia; 4 Department of Toxicology, Universidad de La Laguna, La LagunaCanary Islands, Tenerife, Spain

**Keywords:** Dental fluorosis, Fluoride, Water, Risk assessment, Ion-selective electrode

## Abstract

Fluoride concentrations of 0.5 mg/L in drinking water are considered useful for dental caries prevention. However, fluoride concentrations higher than 1.2 mg/L in water can pose a risk of dental fluorosis due to high exposure to fluoride. The objective is to determine the fluoride concentration in water from aqueducts of different Colombian municipalities of Cauca (Popayán, Coconuco, and Puracé) to assess the fluoride dietary intake from the consumption of this water. A total of 66 water samples have been taken from Popayán, Coconuco, and Puracé. Fluoride content was determined by fluoride ion-selective electrode (ISE) potentiometry. The fluoride concentrations recorded in Coconuco and Puracé water were ≤ 0.002 mg/L. The mean fluoride content recorded in the Popayán water was 0.42 mg/L, with its highest concentration in Cauca River water (0.83 mg/L). Considering the admissible intake values, the water from Popayán confers remarkable fluoride intakes, especially in children with high percentages of contribution to the admissible daily intake (46.7% to 7- to 12-month children and 41.5% to 1- to 3-year children). The fluoride content in the water of Coconuco and Puracé does not reach an optimal value (< 0.5 mg/L) for the protective effect against dental caries, while the water of the main Cauca River basin does reach the optimal value. Likewise, the intake of fluoride from the consumption of the analyzed water does not confer any health risk. However, the implementation of monitoring systems for fluoride levels is recommended in order to safeguard the consumer’s health.

## Introduction

Dental fluorosis is an enamel development defect (DDE) caused by a high intake of fluorides, which generally come from drinking water (Mandinic et al., 2010). It is considered an endemic condition in areas where the water has concentrations that exceed 1.5 mg/L (MINSALUD, 2016a, b). This defect has its origin during the formation of dental tissues, between 20 and 36 months of age for permanent dentition and during intrauterine life for primary dentition, although in the latter, fluorosis is less frequent (Azpeitia-Valadez et al., 2008).

When fluoride is incorporated into the structure of dental enamel during the formation stage, alterations can be generated in the transport of ameloblasts and in the intracellular degradation of matrix proteins, with the consequent delay in the elimination of proteins, mainly of amelogenins (Castiblanco et al., 2017; EFSA, 2013). This process prevents the thickening of the hydroxyapatite crystals and leads to incomplete mineralization. In addition, kallikrein, responsible for the reabsorption of the organic part, is inhibited. It is for this reason that mineralization is not carried out at the respective times and in the quantities that are needed; as a result, there will be an adamantine structure with weak crystals (Castiblanco et al., 2017).

In the process of amelogenesis, the ameloblasts or enamel-forming cells are required to transport minerals (HPO_2_, CO_2_, Na^+^, F^−^) and amino acids from the plasma into the cell, to give rise to the enamel proteins, send them to the extracellular matrix and form hydroxyapatite crystals (Castiblanco et al., 2017; Revelo-Mejía et al., 2020). The main proteins that participate in this process are amelogenin, ameloblastin, enamelin, and tufthelin, called matrix metalloproteins like MMP-20 and kallikrein (KLK4). MMP-20 degrade proteins in the secretion and maturation stage, but at the beginning of maturation, they stop being produced and begin to form KLK4 that modifies the enamel protein matrix, remodels the organic zone so that this space is occupied by the inorganic part and hydroxyapatite crystals are thicker (EFSA, 2013; Rivas-Gutiérrez & Huerta-Vega, 2005).

At the macroscopic level, anomalies are observed in the enamel subsurface that is characterized by increased permeability and the generation of opaque whitish stains, with striations or transverse fissures that are transparent on the surface of the dental enamel (Hardisson et al., 2003; Sukhabogi et al., 2014). The presence of pits with hypomineralized areas can be observed, which form pigmentations over time to form brown spots that can cause fractures and alteration of dental morphology with the appearance of other pathologies such as dental caries mainly (Agudelo-Suárez et al., 2013; Irigoyen-Camacho et al., 2016; Jáudenes-Marrero et al., 2015; Martinez-Mier et al., 2016).

For a definitive diagnosis of dental fluorosis to be made, by means of enamel anomalies, evident at a clinical level, it is necessary to start from the premise that fluoride must be present in drinking water in concentrations greater than 1 mg/L. This clarification should be taken into account since there are several indices, such as the Dean and Leabell (DL) 1945 and the Thylstrup and Fejerskov (TF) of 1978, which were developed by their authors to describe the clinical characteristics, define the severity according to stages and decide the indicated therapy, but not to diagnose the pathology. Currently, there are other mechanisms to classify lesions caused by fluorosis, such as transillumination used in superficial and deep lesions with measurements of approximately 30 μ (Espinosa-Fernández et al., 2012; Strassler & Pitel, 2014).

In river water, there are minerals that come from the riverbed through which they pass, so they can contain phenols, arsenic (As), lead (Pb), selenium (Se), and iron (Fe), generally from industrial waste, in addition, copper (Cu), zinc (Zn), magnesium (Mg), chlorides (Cl^−^), sulfates (SO_4_^2−^), calcium (Ca), iodine (I), nitrates (NO_3_^−^), and you can also find fluorides, although more scarce, originated from ash and igneous or volcanic origin rocks (Hardisson et al., 2003; Jadhav et al., 2015; Paz et al., 2017).

Water can have fluoride in varying concentrations. Water resources located in mountainous areas or in areas with geological deposits of marine origin have a higher concentration. Seawater contains fluoride between 0.8 and 1.4 mg/L, and freshwater shows large oscillations, generally in the form of alkaline fluorides (Concha-Loaiza, 2012). As for the admissible value of fluoride in force for Colombia, it is 1.2 mg/L set in National Decree 1575 of 2007 (MINSALUD, 2007).

**Fig. 1:**
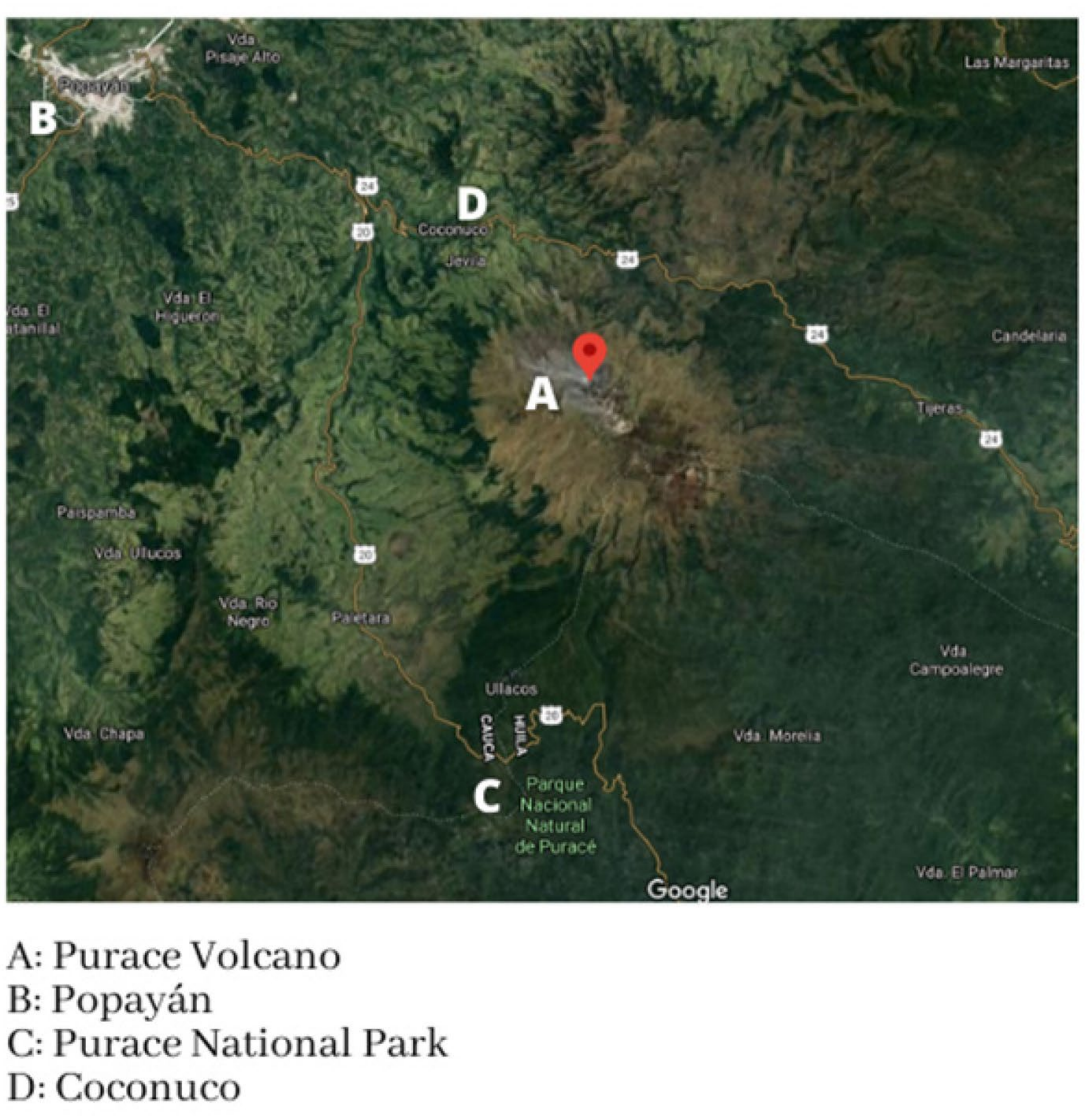
Geographical area of the sampling points

The hydrographic grid of the central area of the department of Cauca, where the municipalities of Popayán, Coconuco, and Puracé are located, is highly diversified, due to the heterogeneity of the relief and the configuration of the mountain system, with various depressions and valleys that determine that the drainage of water move in a distributive sense due to the large differences in height they present. The rivers generally run through deep canyons due to the steep forms of the relief. The soils of the region vary (Fig. 1) moderately from medium to deep and are mostly clayey in textures (CRC, 2012).

The geographical location of Popayán corresponds to the Central Cordillera of the Andes (Goyes-Peñafiel & Hernandez-Rojas, 2021). It is related to the Colombian Massif, to the Puracé volcanic area, and to the nearby presence of hot springs (Maldonado et al., 2017; Torres-Ceron et al., 2019). These characteristics suggest that the mineral content in these sources may be high (Fig. 1).

The municipality of Coconuco and Puracé is politically only one, but geographically it has two headwaters and offers great water wealth (Fig. 1) since important rivers and streams that belong to the great Cauca-Magdalena basin are born in its territory (IDRNR, 1972; Londoño-Arango, 2001; Rodríguez et al., 2018). The most important geological feature of this municipality is related to the post-glacial activity of the Puracé volcano. The subsoil is made up of metamorphic rocks from the Paleozoic era, igneous rocks from the Triassic-Jurassic period, sedimentary rocks from the Cretaceous age, and to a greater extent, Tertiary-Quaternary volcanic rocks (Cooper et al., 1995; Rodríguez-García et al., 2020; Schamel, 1991).

It is necessary to monitor the levels of fluoride in the water resources of the different regions of Colombia in order to obtain current data and detect possible high levels of fluoride that may pose a risk to the health of the inhabitants of that region. Likewise, it will be determined if the fluoride content in the analyzed water reaches an optimal value to offer protection against dental caries.

The objective of this study is to determine the fluoride content in surface river water in the regions of Popayán, Coconuco, and Puracé and to evaluate the dietary intake of fluoride from the consumption of that water in the different population groups.

## Material and methods

### Samples

A total of 66 water samples were taken from the micro-basins that supply the main urban and rural aqueducts in the central area of the department of Cauca (Colombia) (Table 1).

**Table 1:** Samples collected and sampling points

Region	Sampling point	No. of samples
Popayán	Cauca river	9
	Las Piedras river	9
	Molino river	9
	Palacé river	9
	Pisojé river	9
Coconuco	Coconuco river	6
	Coconuco and Tinajuela rivers union	6
Puracé	Puracé river	9

**Fig. 2:**
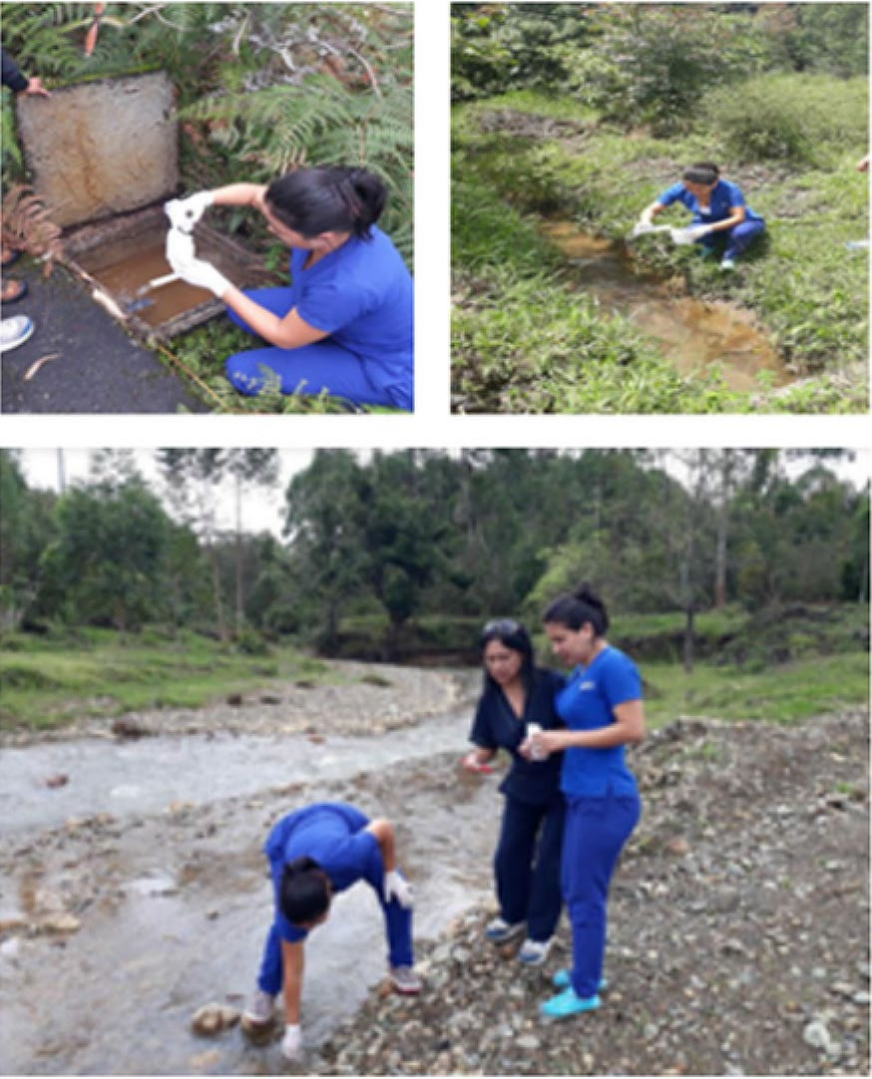
Sample of the water sample collection process

The water was collected directly from the rivers, at the confluence of two rivers, intakes, storage tanks, and residential water (Fig. 2). It was stored in polyurethane containers with the respective biosafety standards. The protocol established by the Ministry of Social Protection has been followed according to the requirements set forth in Article 27 of Decree 1575 of 2007 (MINSALUD, 2007). The samples were labeled and refrigerated at 4 °C until their analysis (Zhu et al., 2013).

### Analytical method

The determination of fluoride was carried out by potentiometry with a selective fluoride ion electrode (CRISON, Barcelona, Spain) (Jaudenes et al., 2018; Jáudenes-Marrero et al., 2015; Paz et al., 2017).

This electrochemical technique uses a solid-state half-cell electrode, whose main component is a laser-like coated crystal, with a lanthanum fluoride (LaF_3_) membrane bonded to an epoxy body. When the electrode is immersed in a water sample containing fluoride ions, an electrical current is generated between the sample and the internal solution of the electrode, creating an electrolytic cell potential, through which only fluoride ions (F^−^), which are recorded in the form of ionic activity or concentration (Jáudenes-Marrero et al., 2015).

A calibration curve was performed with solutions of known fluoride concentration (10^−1^, 10^−2^, 10^−3^, 10^−4^, 10^−5^ M) (Rodríguez-Gómez et al., 2003; Rubio et al., 2020; Revelo-Mejía et al., 2021). The potential of the samples was measured, and the concentration was extrapolated using the semi-log calibration curve prepared above. The samples and the prepared solutions were previously conditioned with orthophosphoric acid (0.75 M) (Sigma-Aldrich, Germany) to adjust the ionic strength and pH (Rodríguez et al. 2018; Rubio et al., 2020).

### Dietary intake calculations

The dietary intake assessment was conducted by calculating the estimated daily intake (EDI) (Eq. 1) and the contribution percentage (Eq. 2) considering the values of daily tolerable upper intake levels (ULs) of fluoride (Table 2) established for each age group by the Food and Nutrition Board of the Institute of Medicine (IOM, 2004). Considering a water consumption of 0.6 L/day (7 − 12 months), 0.9 L/day (1 − 3 years), 1.2 L/day (4 − 8 years), 1.8 L/day (9- to 13-year men), 1.6 (9- to 13-year women), 2.6 L/day (14- to 18-year men), 1.8 L/day (14- to 18-year women), 2.2 L/day (women; 19 − 70 + years), and 2.6 L/day (men; 19 − 70 + years) for the Colombian population (MINSALUD, 2016a, b).

**Table 2:** Daily tolerable upper intake levels (ULs) for fluoride by age groups set by IOM (2004)

Age	Genre		Physiological state	
	Men	Women	Pregnancy	Lactation
Birth to 6 months	0.7 mg	0.7 mg	-	-
7–12 months	0.9 mg	0.9 mg		
1–3 years	1.3 mg	1.3 mg		
4–8 years	2.2 mg	2.2 mg		
9–13 years	10 mg	10 mg		
14–18 years	10 mg	10 mg	10 mg	10 mg
19–51 years	10 mg	10 mg	10 mg	10 mg
51 + years	10 mg	10 mg	-	-

1 $$\begin{aligned}EDI (\mathrm{mg}/\mathrm{day}) &= Fluoride\;concentration\;(\mathrm{mg}/\mathrm{L})\\& \times Water\;consumption\; (\mathrm{L}/\mathrm{day})\end{aligned}$$

2 $${Contribution }\;(\mathrm{\%}) = [EDI / ULs] \times 100$$

### Statistical analysis

Statistical analysis was performed using GraphPad Prism 8.4.3 software (GraphPad Software, Inc., California, USA) for Windows™.

The distribution of the data has been studied by applying the one-way ANOVA. The Welch’s test was used to study the existence of statistical differences (*p* < 0.05) in the fluoride content between the analyzed areas (Popayán, Cononuco, and Puracé) (Yap & Sim, 2011).

## Results and discussion

### Fluoride concentration in water from Popayán, Coconuco, and Puracé

In the water samples taken in the municipality of Coconuco, values equal to and/or less than 0.002 mg/L (LOD, limit of detection) were recorded (Table 3). On the other hand, in the analysis of the water samples from the rivers of Puracé, the values found were equal to and/or lower than 0.002 mg/L (LOD) (Table 3).

**Table 3:** Concentration (mg/L) of fluoride content in Popayán, Coconuco, and Puracé

Region	Water source	Sampling point	Concentration (mg/L)	Min–max value (mg/L)
Popayán	Piedras river intake	Vereda Santa Helena	0.002 ± 0.001	< DL–0.002
	Palacé river intake	Vereda Santa Helena	0.002 ± 0.001	< DL–0.002
	Molino river intake	Vereda Santa Bárbara	0.002 ± 0.001	< DL–0.002
	Pisojé river intake	Vereda Vinagre	0.002 ± 0.001	< DL–0.002
	Cauca river intake	Vereda El Carmen	0.83 ± 0.005	0.82–0.83
	Mean		0.42 ± 0.44	< DL–0.83
Coconuco	Coconuco-Tinajuela river	River source	0.002 ± 0.001	< DL–0.002
	Coconuco-Tinajuela river	River source (confluence)	0.002 ± 0.001	< DL–0.002
	Coconuco river	Storage tank	0.002 ± 0.001	< DL–0.002
	Coconuco river	Residential water	0.002 ± 0.001	< DL–0.002
	Mean		0.002 ± 0.001	< DL–0.002
Puracé	Puracé river	Piedra Grande source	0.002 ± 0.001	< DL–0.002
	Puracé river	Storage tank	0.002 ± 0.001	< DL–0.002
	Puracé river	Residential water	0.002 ± 0.001	< DL–0.002
	Mean		0.002 ± 0.001	< DL–0.002

DL (detection limit) of 0.001 mg/L

**Fig. 3:**
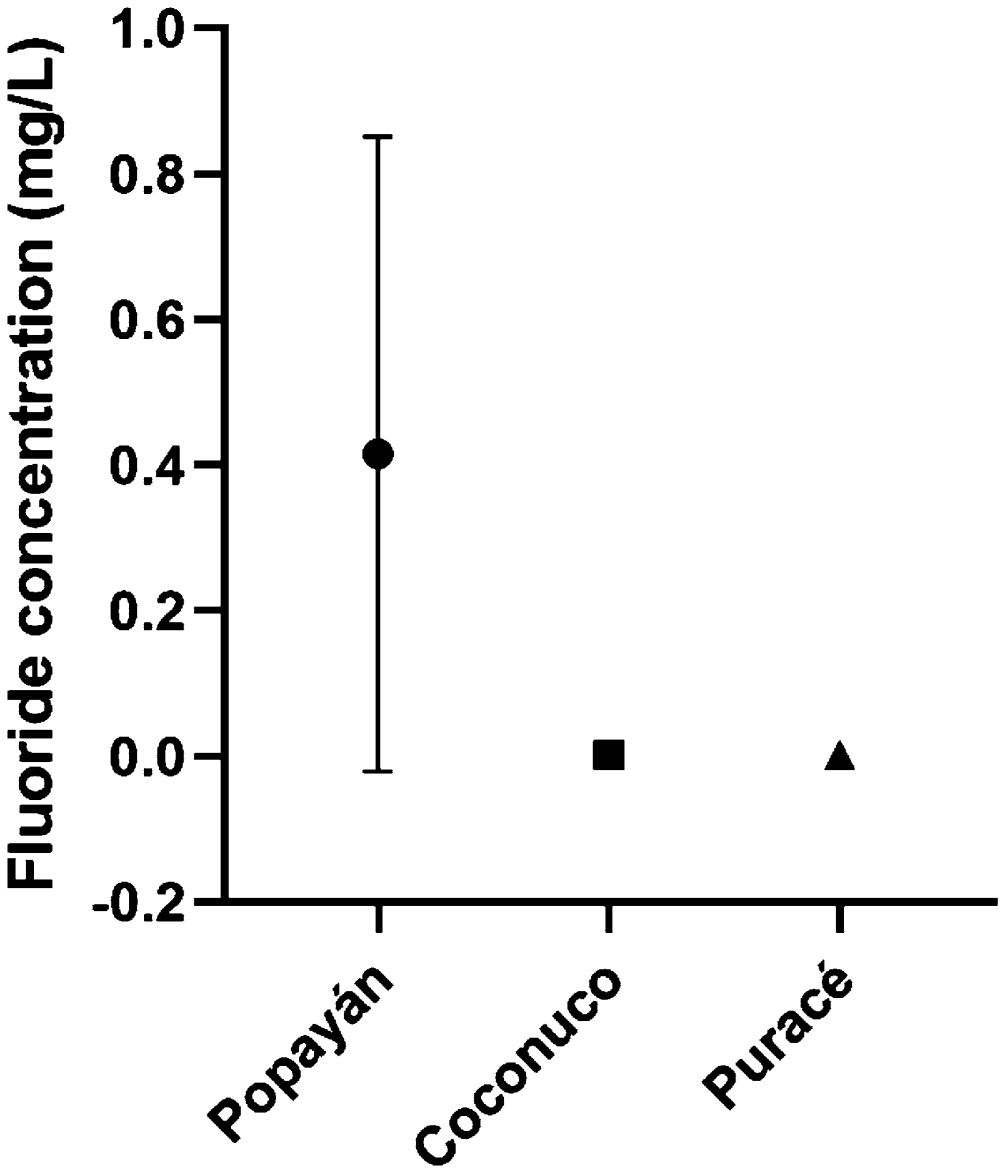
*Comparison* of the fluoride concentration between different sampling areas

Fluoride concentrations in water from the Popayán aqueducts (Table 3) from the four sub-basins (Piedras, Palacé, Molino, and Pisojé river intake) are less than 0.5 mg/L, finding concentrations less than or equal to the detection limit (LD) of 0.002 mg/L. In the water of the four sub-basins Piedras, Molino, Palacé, and Pisojé, which supply the urban and rural aqueducts of the Municipality of Popayán, insufficient concentrations were recorded to prevent dental caries, that is, they did not reach the optimum minimum indicated in 0.5 mg/L. Figure 3 indicates the comparison of the fluoride content in the water of Popayán, Coconuco, and Puracé.

Regarding the fluoride content in the great basin (Cauca River), an average concentration of 0.83 mg/L was recorded. This concentration registered in the Cauca River does confer a beneficial effect since it reaches an optimal level to achieve the protective effect of dental caries. In addition, it does not represent a risk of dental fluorosis since it does not exceed the parameter of 1.2 mg/L and, according to the literature, does not reach to interfere in the amelogenesis process (Browne et al., 2005; Ji et al., 2018; Khalaf, 2021).

Only this last finding verifies the theory of the presence of fluorides in waters near volcanic areas, where the content of fluoride in water depends on the presence of fluorinated minerals in natural sources and on the existence of atmospheric pollutants resulting from the emanation of Volcano gases (Battaleb-Looie et al., 2012; D’Alessandro, 2006; Petrone et al., 2013; Rango et al., 2012). This coincides with the data reported from the micro-basins that surround the Galeras Volcano, specifically the Río Azufral and Río Cariaco located in the municipality of Consacá (Nariño) with similar concentrations of 0.8 mg/L (Guerrero-Burbano et al., 2008). In the case of Pasto due to the Galeras Volcano and in the case of Popayán due to its proximity to the Puracé volcano.

Based on the National Decree 1575 of 2007 of the Ministry of Social Protection of Colombia (MINSALUD, 2007) regarding fluoride concentrations, it can be shown that the physicochemical analysis of the water sources belonging to the municipality of Coconúco and Puracé yielded a result according to the reference values and within the range, without risk for human consumption because all the values found were less than 0.5 mg/L. With the exception of a single site, the water from the Cauca River with 0.83 mg of fluoride per liter, the other samples were taken in the remaining areas registered fluoride levels lower than 0.5 mg/L. According to the fluoride concentrations recorded, the analyzed water is not useful to prevent dental caries and does not pose a risk for dental or bone fluorosis (Castro et al., 2014).

However, these findings do not completely exempt the risk of suffering from this type of pathology. It is also necessary to take into account that there are various sources of water supply, such as wells, especially in rural areas, that can be summative between them. The mineral contents of these sources are high considering that the greater or lesser amount of fluorides depends on the depth, porosity of the rocks, soil, temperature, and the concentration of hydrogen and calcium ions present in the water. In addition, it is known that alkaline waters and those with a high temperature are more likely to have higher concentrations of fluoride (EFSA, 2013; Lin et al., 2016).

### Statistical study of the fluoride concentration

Table 4 indicates the statistical parameters studied and the results obtained. Through the application of the one-way ANOVA test, the normal distribution of the data from the different study areas is demonstrated.

**Table 4:** Statistical study and parameters

Statistical parameters	Puracé vs Popayán	Coconuco vs Popayán	Coconuco vs Puracé^a^
Unpaired *t*-test with Welch’s correction			
*P*-value	0.1747	0.1747	ns
*P*-value summary	ns	Ns	ns
Significantly different (*P* < 0.05)	No	No	ns
One- or two-tailed *P*-value?	Two-tailed	Two-tailed	ns
Welch-corrected *t*, df	*T* = 1.581 df = 5.000	*T* = 1.581 df = 5.000	ns
*F*-test to compare variances			
F, DFn, Dfd	Infinity, 5, 2	Infinity, 5, 3	ns
*P*-value	< 0.0001	< 0.0001	ns
*P*-value summary	****	****	ns
Significantly different (*P* < 0.05)?	Yes	Yes	ns

^a^Since all the values in both columns are identical, it is not possible to compute a *t*-test

Then, applying the Welch’s test, the nonexistence of significant differences (*p* > 0.05) in the fluoride content in the different areas studied (Popayán, Coconuco, and Puracé) was confirmed. However, significant differences (*p* < 0.05) were recorded between the variances of each area. This means that the variances were not distributed homogeneously. This is due to the data discrepancy recorded in the Popayán area (samples collected from the Cauca River in Vereda El Carmen).

### Comparison with other authors

Table 5 shows fluoride concentration values in other studies carried out by other authors. It is noteworthy that, in volcanic areas of the Canary Islands (Spain), the concentrations of fluoride registered in the water are much higher (4.22 mg/L in water from Tenerife) compared to those registered in the present study. It should be noted that the supply water of the island of Tenerife comes entirely from underground galleries and wells, which is why the fluoride content is higher since the water is in contact for a longer period of time with minerals than contain fluoride.

**Table 5:** Comparison of fluoride content in water from different geographical areas

Area	Year	Fluoride concentration (mg/L)	Reference
Popayán, Colombia	2021	0.42 ± 0.44	Present study
Coconuco, Colombia		0.002 ± 0.001	
Puracé, Colombia		0.002 ± 0.001	
Timbío, Colombia	2020	0.121–0.210	Revelo-Mejía et al. (2020)
Santander de Quilichao, Colombia		0.027–0.068	
Tenerife, Canary Islands, Spain	2020	4.22 ± 2.11	Rubio et al. (2020)
El Hierro, Canary Islands, Spain		0.36 ± 0.29	
La Palma, Canary Islands, Spain		0.38 ± 0.12	
Zimbabwe	2016	> 1.5	Kut et al. (2016)
Tanzania		> 45	
Mongolia, China	2015	1.59	Jadhav et al. (2015)
Santiago del Estero, Argentina		0.7–22	

On the other hand, in water from other regions of Colombia, higher concentrations of fluoride have been recorded, as is the case of water from Timbío (0.121–0.2010 mg/L). In other areas of South America, such as Argentina, Jadhav et al. (2015) recorded fluoride concentrations in water that ranged between 0.7 and 22 mg/L, also being higher than those recorded in the present study.

In other geographic regions such as Zimbabwe or Tanzania, the fluoride concentrations recorded are higher than those of the present study, highlighting the case of Tanzania with a fluoride concentration higher than 45 mg/L. It is known that the area of Tanzania stood out for its prevalence of dental fluorosis for quite some time (Yoder et al., 1998) due to the presence of the mineral trona or Magadi (Kaseva, 2006).

It is evident that the fluoride content in water depends on various factors, which is why studies to determine fluoride levels in drinking water are necessary to know the current state of the water, especially in impoverished rural areas of Colombia, those where the water of public supply is of vital importance.

### Dietary intake assessment

Table 6 shows the estimated daily intakes (EDI) and the percentages of contribution to the established ULs (IOM, 2004). The evaluation of fluoride intake has been carried out considering the recommended consumption of water established by the Colombian authorities (MINSALUD, 2016a, b).

**Table 6:** Fluoride intake (mg/day) from the consumption of the analyzed Colombian water and percentage of contribution to the UL values

Age	ULs (mg/day)	EDI (mg/day)			Contribution (%)		
		Popayán	Coconuco	Puracé	Popayán	Coconuco	Puracé
7–12 months	0.9	0.25	0.0012	0.0012	27.8	0.13	0.13
1–3 years	1.3	0.54	0.003	0.003	41.5	0.23	0.23
4–8 years	2.2	0.50	0.0024	0.0024	22.7	0.11	0.11
9–13 years	10	0.67^a^–0.76^b^	0.0032^a^–0.0036^b^	0.0032^a^–0.0036^b^	6.7^a^–7.6^b^	0.03^a^–0.04^b^	0.03^a^–0.04^b^
14–18 years	10	0.76^a^–1.10^b^	0.0036^a^–0.0052^b^	0.0036^a^–0.0052^b^	7.6^a^–11.0^b^	0.04^a^–0.05^b^	0.04^a^–0.05^b^
19–51 years	10	0.92^a^–1.10^b^	0.004^a^–0.005^b^	0.004^a^–0.005^b^	8.30^a^–10.4^b^	0.04^a^–0.05^b^	0.04^a^–0.05^b^
51 + years	10						

Considering a water consumption: 0.6 L/day (7–12 months), 0.9 L/day (1–3 years), 1.2 L/day (4–8 years), 1.8 L/day (9- to 13-year men), 1.6 (9- to 13-year women), 2.6 L/day (14- to 18-year men), 1.8 L/day (14- to 18-year women), 2.2 L/day (women; 19–70 + years), and 2.6 L/day (men; 19–70 + years) (MINSALUD, 2016a, b)

^a^Women

^b^men

Consuming the water from Popayán offers estimated daily fluoride intakes of 0.92 mg/day for adult women and 1.10 mg/day for adult men. In the case of the child population, fluoride intake ranges from 0.25 mg/day (infants 7 to 12 months) and 0.76 mg/day (male children 9 to 13 years).

The percentage of contribution to the UL from the consumption of water from Popayán in the child population aged 1 to 3 years stands out, with a percentage of 41.5% over the maximum value established at 1.3 mg/day by the IOM (2004). Although it does not exceed the maximum value, it does reach a notorious percentage, especially if we consider that water is not the only dietary source of fluoride in humans, and probably the population between 1 and 3 years of age will consume other types of products rich in fluorides such as juices, juices, soft drinks, etc. Therefore, in these cases, consumption of bottled water could be recommended since the global intake of fluoride could exceed the maximum limit established with the consequent risk to health.

In general terms, the present study does not suggest an alarm for general and dental health. This does not mean that it should be neglected and not carried out through continuous monitoring of both the water sources and the population that nourishes them (MINSALUD, 2015) therefore, although there is no considerable report of cases with dental fluorosis, there have been many cases diagnosed with presumptive diagnoses of this pathology reported in the university dental clinic of Popayán (Revelo-Mejía et al. 2020).

There are studies that prove the existence of a direct association between the natural concentration of fluorides in drinking water and the presence of dental fluorosis. In addition to an inverse association between the concentration of fluoride in the water and the prevalence of dental caries, that is, as the concentration of fluoride in the water increases (> 1.0 mg/L), the number of carious lesions in the permanent dentition and the prevalence of dental fluorosis increases (Beltrán-Aguilar et al., 2010).

It is key then to note that water consumption is the main source to trigger dental fluorosis. Other factors such as food, rinses, and toothpaste are ruled out because consumption does not reach the necessary concentrations to interfere with the formation of enamel in the age of mineralization (Celik et al., 2013). Therefore, it is better to make an excellent diagnosis and rule out the wide variety of differential diagnoses described by dental pathology (Revelo-Mejía et al. 2020).

## Conclusions

The concentrations of natural fluoride found in the water of the four sub-basins that supply the urban and rural aqueducts of the Popayán municipality, as well as the water sources that feed the Coconuco and Puracé aqueducts, are below the parametric value established in the Colombian regulations and, therefore, the water is safe for human consumption. The statistical analysis confirms the normal distribution of the data and the nonexistence of significant differences (*p* > 0.05) in the fluoride content in the different areas studied (Popayán, Coconuco, and Puracé). But, significant differences (*p* < 0.05) were found between the variances of each area. This is due to the data discrepancy recorded in the Popayán area from the Cauca River in Vereda El Carmen.

On the other hand, the fluoride content in the analyzed water is below the optimal value to offer protection against dental caries, with the exception of the fluoride content registered in one of the basins of the municipality of Popayán (El Carmen village), whose registered fluoride concentration is sufficient to prevent dental caries and, furthermore, does not exceed the permissible limit value established in Colombian legislation.

Although the analyzed water does not pose a risk to the health of consumers, it is necessary to establish monitoring programs for the fluoride content in the water in order to preserve the health of consumers.
